# Circulating adipose-tissue miRNAs in gastrointestinal cancer patients and their association with the level and type of adiposity at body composition analysis

**DOI:** 10.3389/fmolb.2024.1449197

**Published:** 2024-07-30

**Authors:** Federica Tambaro, Giovanni Imbimbo, Valentina Pace, Maria Ida Amabile, Veronica Rizzo, Simona Orlando, Giulia Lauteri, Cesarina Ramaccini, Carlo Catalano, Giuseppe Nigri, Maurizio Muscaritoli, Alessio Molfino

**Affiliations:** ^1^ Deparment of Translational and Precision Medicine, Sapienza University of Rome, Rome, Italy; ^2^ Department of Surgery, Sapienza University of Rome, Rome, Italy; ^3^ Department of Radiological, Oncological and Pathological Sciences, Sapienza University of Rome, Rome, Italy; ^4^ Department of Medical and Surgical Sciences and Translational Medicine, Sapienza University of Rome, Rome, Italy

**Keywords:** adiposity, miRNAs, cachexia, cancer, CT-scan, body composition

## Abstract

**Background:**

Adipose tissue (AT) wasting in cancer is an early catabolic event with negative impact on outcomes. Circulating miRNAs may promote body weight loss and cachexia. We measured circulating miRNAs linked to AT alterations and compared their levels between i) gastrointestinal (GI) cancer patients and controls, ii) cachectic and non-cachectic cancer patients, and iii) according to adiposity level and its distribution.

**Methods:**

Patients with GI cancer and subjects with benign diseases as controls were considered. Cachexia was assessed and adiposity evaluated by CT-scan for subcutaneous AT area (SAT), visceral AT area and the total AT area (TAT). MiRNAs involved were measured in plasma by RT-qPCR.

**Results:**

37 naïve GI cancer patients and 14 controls were enrolled. Patients with cachexia presented with lower SAT compared to non-cachectic (*p* < 0.05). In cancer patients, we found higher levels of miR-26a, miR-128, miR-155 and miR-181a vs. controls (*p* < 0.05). Cancer patients with BMI < 25 kg/m^2^ showed higher levels of miR-26a vs. those with BMI ≥ 25 (*p* = 0.035). MiR-26a and miR-181a were higher in cachectic and non-cachectic vs. controls (*p* < 0.05). Differences between cachectic and controls were confirmed for miR-155 (*p* < 0.001) but not between non-cachectic vs. control (*p* = 0.072). MiR-155 was higher in cachectic patients with low TAT vs. those without cachexia and high TAT (*p* = 0.036).

**Conclusion:**

Our data confirm a modulation of specific and different miRNAs involved in AT metabolism in cancer and cachexia. MiR-155 levels were higher in patients presenting with cachexia and low adiposity with implications in the pathogenic mechanisms and clinical consequences of GI cancer patients.

## 1 Introduction

In the last years, adipose tissue derangements were indicated as active promoters of cachexia in cancer patients, with potential negative impact on patient’s survival (Al-Sawaf et al., 2023). Strong evidence showed that adipose tissue wasting often precedes the clinical diagnosis of cancer representing an early event during the disease (Babic et al., 2023). Importantly, adipose tissue has several functions other than energy storing, including release of endocrine hormones, promoting inflammation and regulating the expression of different genes, as well as energy expenditure (Cypess, 2022). In parallel, modifications in terms of quantity and quality of adipose tissue were found in the cancer cachexia phenotype (Molfino et al., 2023a). Recently, data showed that patients with gastrointestinal cancer undergoing surgery for cancer resection presented deep histomorphological alterations of adipose tissue, as well as changes in molecular markers of browning and lipolysis (Molfino et al., 2022a; Molfino et al., 2023a; Tambaro et al., 2024). Also, Taylor J et al. (2023) evaluated the cancer capacity to manipulate the endothelium in white adipose tissue and found that the intracellular pathway signaling promoting adipose tissue wasting was modulated. In this light, cancer-released factors are promising agents to target for the treatment of cancer cachexia. Among them, miRNAs were extensively studied for their capacity to modulate gene expression. Also, miRNAs may act distally being transported in exosomes as endocrine factors and possibly serving as biomarkers in some clinical settings, including cancer (Belli et al., 2021). Importantly, Tomou T et al. showed that adipose tissue represents an important source of circulating exosomal miRNAs, acting as adipokines (Thomou et al., 2017).

In cancer, some miRNAs were found to be potentially implicated in adipose tissue metabolism and wasting. Recently, among others, miR-26a-5p, miR-128, miR-144, miR-181a-5p, and miR-155 were identified as potential regulator of adipose tissue metabolism, such as lipolysis, browning of WAT, and adipogenesis (Tao et al., 2015; Chen et al., 2018; Kim et al., 2020; Hongfang et al., 2023), all phenomena deeply involved in the development of cancer cachexia and therefore of interest in the setting of gastrointestinal cancer.

However, data on the association between these miRNAs and cancer cachexia and adipose tissue wasting in humans are still scanty or lacking.

For this reason, by this study we aimed to evaluate differences in specific circulating miRNAs profile, linked to adipose tissue alterations between i) cancer patients and controls, ii) cachectic and non-cachectic cancer patients, and iii) according to adiposity level (low or high) and distribution assessed by CT-scan.

## 2 Materials and methods

### 2.1 Study design and participant’s enrollment

We conducted a cross-sectional study on patients with a new diagnosis of gastrointestinal cancer (gastric, colon and pancreatic cancer), known to be frequently associated with cachexia (Baracos et al., 2018), eligible for surgical tumor resection, and controls undergoing surgery for non-malignant diseases. The enrollment was carried out at the Department of Medical and Surgical Science of Sapienza University of Rome, Italy. The experiments were conducted at the Department of Translational and Precision Medicine of Sapienza University of Rome, Italy.

The inclusion criteria of this study were age ≥18 years; recent diagnosis of cancer (≤4 weeks); not having received anticancer or anti-inflammatory treatments before surgery; the ability to provide signed informed consent. Exclusion criteria were the presence of coexisting conditions inducing malnutrition such as infections, liver failure, renal failure, heart failure, rheumatologic disorders, clear signs of malabsorption or intestinal occlusion, as well as dysphagia. The study was conducted according to the Declaration of Helsinki and approved by the local Ethics Committee (Sapienza University, Azienda Sant’Andrea Hospital, Rome, Italy—prot. n. 167_2017). Written informed consent was obtained by all the participants enrolled in the study.

### 2.2 Clinical parameters, nutritional status and body composition analysis for adiposity

At first study visit, we collected demographic information and patient’s medical history, as well as data on the staging and histology of the cancer. We registered body weight (kg) and height (m), calculated the body mass index (BMI, kg/m^2^) and asked for usual weight and unvoluntary body weight loss in the previous 6 months. Cachexia was diagnosed according to the Fearon’s criteria (Fearon et al., 2011).

In fasting condition, we obtained blood samples in EDTA tubes to measure inflammatory and nutritional biomarkers, such as serum C-reactive protein (CRP) and albumin with standard laboratory techniques.

Using CT scans, we measured the abdominal fat area at the level of the third lumbar vertebra (L3), quantifying both the total adipose tissue (TAT) and visceral adipose tissue (VAT), as previously shown (Erdem et al., 2019; Molfino et al., 2022b). The SAT area at the same level was calculated, subtracting VAT from TAT. We defined high or low level of adiposity based on the median values of SAT, VAT and TAT (over the median: high; below the median: low). This approach was previously implemented in the setting of adipose tissue wasting (Abdallah et al., 2023).

OsiriX Lite (v11.0.3, Bernex, Switzerland) was used for the abdominal fat composition analysis.

### 2.3 Plasma samples collection

To analyze circulating miRNAs profiles, fasting blood samples were collected prior to the surgical procedure in EDTA Vacutainer® tubes and centrifuged at 3,000 rpm for 15 min at 4°C. Supernatant plasma was immediately aliquoted into cryovials and stored at −80°C until further analysis.

### 2.4 Extraction, quantification and pre-amplification of circulating miRNAs

Total RNA was extracted from plasma samples (250 μL/sample) using miRNeasy Serum/Plasma Kit (Qiagen, Germantown, MD, United States) according to the manufacturer’s instructions. RNA quantification was performed using Multiskan Sky spectrophotometer (Thermo Fisher Scientific, Waltham, MA, United States). cDNA was synthetized from 10 ng of total RNA using the TaqMan Advanced miRNA cDNA Synthesis kit (Applied Biosystems, Thermo Fisher Scientific, Waltham, MA, United States) following the manufacturer’s instructions.

### 2.5 Analysis of circulating miRNAs profiles

Quantitative real-time PCR was performed with TaqMan Fast Advanced Master Mix (Applied Biosystems, Thermo Fisher Scientific, Waltham, MA, United States), using the QuantStudio® Real Time PCR (Applied Biosystems, Thermo Fisher Scientific, Waltham, MA, United States). Quantification was performed using the following specific TaqMan miRNA Assay (Applied Biosystems, Thermo Fisher Scientific, Waltham, MA, United States) probes: hsa-miR-26a-5p (478926_mir); hsa-miR-128-3p (477892_mir); hsa-miR-144-3p (477913_mir); has-miR-155-3p (477926_mir); hsa-miR-181a-5p (477857_mir). Data were normalized to hsa-miR-16-5p (477860_mir) used as the internal control. Resulting data were analyzed using SDS2.4 Software (Applied Biosystems, Bedford, MA, United States), and fold-change was determined by using the 2^−ΔΔCT^, as previously described (Schmittgen and Livak, 2008). All reactions were performed using three biological replicates and all the experiments were performed in duplicate.

### 2.6 Statistical analyses

Data are presented as the mean ± standard deviation (SD) or standard error of the mean (SEM) and median with interquartile range (25th and 75th percentile) for continuous normally and non-normally distributed variables, as appropriate. The normal/non-normal distribution was assessed by Shapiro-Wilk test. Student’s t-test and One-way ANOVA test or Mann–Whitney test and Kruskal–Wallis test were used for normally distributed data or non-normally distributed data, respectively. The Spearman’s coefficient was used to determine the correlation between miRNA expression and BMI. A *p*-value <0.05 was considered statistically significant. IBM® SPSS Statistics version 26 and Graphpad Prism 5.0 (San Diego, California, United States) were employed in the calculation.

## 3 Results

### 3.1 Clinical characteristics of cancer patients and controls

We enrolled a total of 37 gastrointestinal cancer patients (23 male), affected by colorectal (n = 32; 21 male), gastric (n = 4; 1 male) and pancreatic cancers (n = 1, male). The mean age was 71.6 ± 11.9 years, and BMI (kg/m^2^) was 24.1 ± 3.8.

Control group included 14 patients undergoing surgery for benign diseases (including cholecystectomy, abdominal hernia and cysts), with a mean age of 58.1 ± 14.2 years and a BMI of 25.0 ± 3.3.

Cancer patients were older than controls (*p* = 0.001) and BMI was not different between the two groups. The participant’s characteristics are shown in Table 1.

**TABLE 1:** Participant’s characteristics.

Parameters	Cancer patients (n = 37)	Controls (n = 14)
Age, years^*^	71.6 ± 11.9	58.1 ± 14.2
Male, n (%)	23 (62)	8 (57)
Female, n (%)	14 (38)	6 (43)
Actual weight, kg	70.2 ± 12.6	72.0 ± 11.2
BMI, kg/m^2^	24.1 ± 3.8	25.0 ± 3.3
Cachexia, n (%)	18 (49)	—
Blood parameters		
Hemoglobin, g/dL	12.0 ± 2.1	13.1 ± 1.8
C-reactive protein, mg/dL	4.8 (0.2; 11.7)	1.7 (0.6; 4.7)
Albumin, g/dL	3.4 ± 0.6	3.7 ± 0.4
Type of GI, n (%)		
Colon	32 (87)	—
Gastric	4 (11)	—
Pancreatic	1 (3)	—
Stage of the disease, n (%)		
I-II	24 (65)	—
III-IV	13 (35)	—

Variables are shown as mean ± SD and as median (inter-quartile range) for non-normally distributed values. No differences were shown between cancer patients and controls with exception of age (^*^ *p* = 0.001). Abbreviations: BMI, body mass index.

### 3.2 Prevalence of cachexia and assessment of adiposity by CT-scan

In cancer patients, cachexia was diagnosed in 18 out of 37 cancer patients (49%). Patients with cachexia compared to non-cachectic presented with a decreased BMI (22.8 ± 3.8 vs. 25.3 ± 3.6, *p* = 0.044).

The mean values (cm^2^) of SAT, VAT and TAT in cancer patients were 182.6 ± 72.3, 152.8 ± 86.7 and 335.5 ± 145.2, respectively.

In cachectic patients, we observed lower SAT compared to non-cachectic (157.6 ± 63.7 vs. 206.2 ± 73.5, *p* = 0.039) (Table 2), but no difference was seen in VAT and TAT (Table 2).

**TABLE 2:** Differences between cachectic and non-cachectic cancer patients in clinical and adiposity parameters.

Parameters	Cachexia (n = 18)	No cachexia (n = 19)
Age, years	72.8 ± 12.6	70.4 ± 11.4
Male, n (%)	13 (72)	10 (53)
Female, n (%)	5 (28)	9 (47)
BMI, kg/m^2^^*^	22.8 ± 3.8	25.3 ± 3.6
Stage of the diseases, n (%)		
*I-II*	10 (56)	14 (74)
*III-IV*	8 (44)	5 (26)
Body composition parameters		
SAT, cm^2#^	157.6 ± 63.7	206.2 ± 73.5
VAT, cm^2^	145.8 ± 93.0	159.3 ± 82.3
TAT, cm^2^	303.5 ± 149.1	365.5 ± 138.7

No differences were shown between cachectic and non-cachectic with exception of BMI, and SAT (^*^ *p* = 0.044; #*p* = 0.039). Abbreviations: BMI, body mass index; SAT, subcutaneous adipose tissue area; VAT, visceral adipose tissue area; TAT, total adipose tissue area.

In all cancer patients, BMI strongly and positively correlated with SAT (r = 0.77, *p* < 0.001), VAT (r = 0.64, *p* < 0.001) and TAT (r = 0.74, *p* < 0.001). Other clinical characteristics are shown in Table 2.

### 3.3 Differences in circulating miRNAs between cancer patients and controls

**FIGURE 1:**
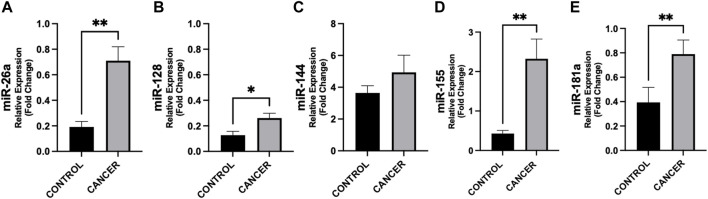
Differences in circulating miRNAs between cancer patients and controls. Circulating miRNAs levels were analyzed by quantitative Real Time PCR from gastrointestinal cancer patients (n = 37) and control group (n = 14). Data show higher expression of miR-26a (*p* = 0.002) **(A)**, −128 (*p* = 0.047) **(B)**, −155 (*p* = 0.004) **(D)**, and −181a (*p* = 0.007) **(E)** in cancer with respect to controls. No difference was observed in miR-144 levels in cancer patients vs. controls **(C)**. Data were normalized against the miR-16 from two biological replicates. ^*^ *p* < 0.05, ^**^ *p* ≤ 0.01, ^***^ *p* ≤ 0.001.

**FIGURE 2:**
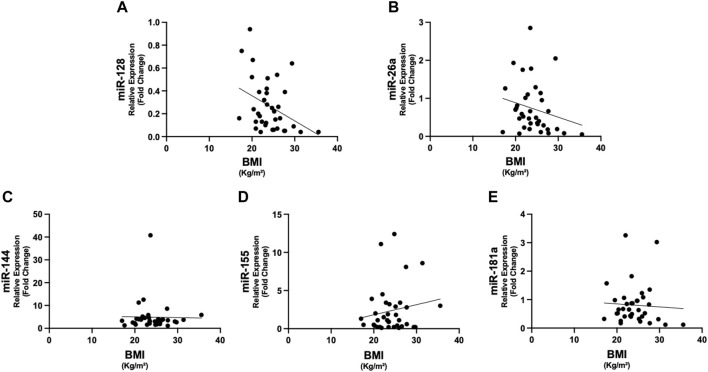
Correlations between circulating miRNAs and BMI in cancer patients. The expression of miR-128 showed negative correlation with BMI (rho = −0.380, *p* = 0.022) **(A)**. No significant correlations were present between the other miRNAs and BMI **(B–E)**. Abbreviations: BMI, body mass index.

In Figure 1, we show the differences in plasma miRNAs between cancer patients and controls. In particular, in patients with cancer we found higher levels of miR-26a, miR-128, miR-155 and miR-181a with respect to controls (*p* < 0.05) (Figure 1). No differences were observed in miR-144 between the two groups (Figure 1). In addition, in cancer patients miR-128 negatively correlated with BMI (rho = −0.380, *p* = 0.022) (Figure 2), whereas no significant correlations were present between BMI and other miRNAs tested (Figure 2). No differences were observed in the miRNAs levels according to cancer stage (data not shown).

**FIGURE 3:**
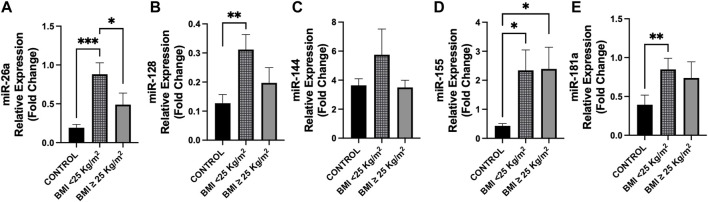
Differences in circulating miRNAs between cancer patients and controls according to BMI classes. Circulating miRNAs levels were analyzed in cancer patients according to BMI (kg/m^2^) classes (BMI < 25, n = 22; BMI ≥ 25, n = 14) compared to control group (n = 14). **(A)** Expression levels of miR-26a were higher in cancer patients with BMI < 25 with respect to those with BMI ≥ 25 (*p* = 0.035) and vs. controls (*p* < 0.001). **(B)** MiR-128 showed overexpression in cancer patients with BMI < 25 vs. controls (*p* = 0.008), whereas no difference was observed with respect to those with BMI ≥ 25 (*p* = 0.071). **(D,E)** MiR-155 and miR-181a levels were higher in cancer patients with BMI < 25 compared to controls (*p* = 0.010 and *p* = 0.003, respectively), whereas miR-155 levels were higher in cancer patients with BMI ≥ 25 vs. controls (*p* = 0.017). **(C)** No differences were observed in miR-144 levels between groups. ^*^ *p* < 0.05, ^**^ *p* ≤ 0.01, ^***^ *p* ≤ 0.001. Abbreviations: BMI, body mass index.

According to BMI classes, patients with BMI < 25 presented with higher plasma levels of miR-26a with respect to cancer patients with BMI ≥ 25 (*p* = 0.035), as well as vs. controls (*p* < 0.001) (Figure 3); a similar trend was observed for miR-128 in cancer group (*p* = 0.071) (Figure 3). No additional differences between BMI classes in cancer patients were observed for the other miRNAs (Figure 3).

### 3.4 Differences in circulating miRNAs among cachectic and non-cachectic cancer patients and controls

**FIGURE 4:**
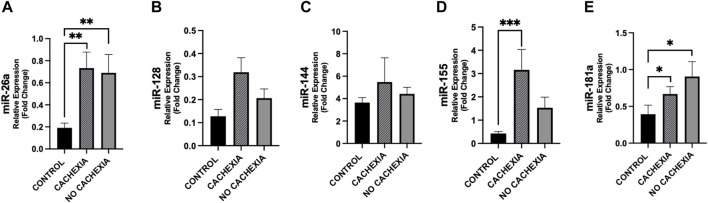
Differences in circulating miRNAs among cachectic and non-cachectic cancer patients and controls. Circulating miRNAs levels were analyzed in cancer patients with cachexia (n = 18), without cachexia (n = 19) and controls (n = 14). Data show higher expression of miR-26a **(A)**, miR-155 **(D)** and miR-181 **(E)** in patients with cachexia (*p* = 0.005, *p* = 0.001 and *p* = 0.023, respectively) with respect to controls and higher expression of miR-26a **(A)**, and miR-181 **(E)** in cancer patients without cachexia (*p* = 0.007 and *p* = 0.012, respectively) with respect to controls. For miR-128, we observed a trend of increased expression in cachectic patients vs. controls (*p* = 0.057) **(B)**. No difference was observed in miR-144 levels in cancer patients with/without cachexia vs. controls **(C)**. ^*^ *p* < 0.05, ^**^ *p* ≤ 0.01, ^***^ *p* ≤ 0.001.

In Figure 4, we show the differences in plasma miRNAs between cancer patients with and without cachexia and controls. In particular, plasma levels of miR-26a resulted higher in cachectic patients (*p* = 0.007), as well as in non-cachectic (*p* = 0.005) vs. controls; this was also observed for miR-181a (*p* < 0.05) (Figure 4).

Differences between cachectic patients and controls were also found for miR-155 (*p* < 0.001) and did not reach the significance between non-cachectic and controls (*p* = 0.072) (Figure 4). Also, miR-155 showed a trend of increase in cachectic compared to non-cachectic patients (*p* = 0.087) (Figure 4). No significant differences were seen in miR-128 and miR-144 between groups (Figure 4).

### 3.5 Differences in circulating miRNAs according to adiposity level and distribution assessed by CT-scan in cachectic and non-cachectic cancer patients

**FIGURE 5:**
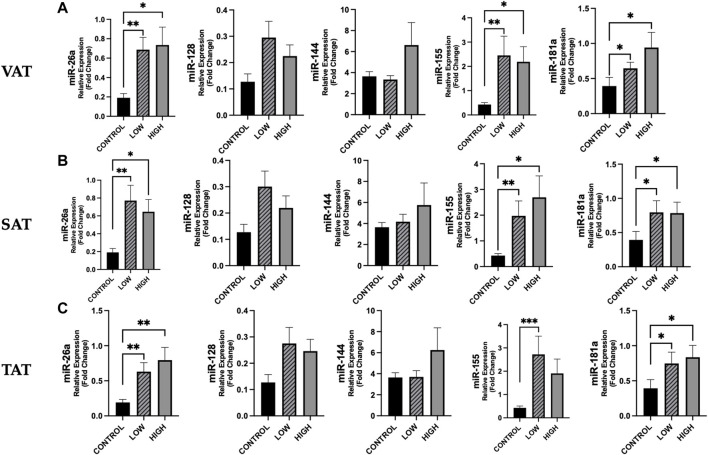
Differences in circulating miRNAs according to adiposity level among cancer patients and in controls. We analyzed the data according to the median level of adiposity, as follows: low (n = 19) vs. high (n = 18) of VAT (median: 130.70 cm^2^), SAT (median: 167.57 cm^2^), and TAT (median: 322.56 cm^2^) and in controls (n = 14). **[(A)**, from left to right**]** Data show higher expression of miR-26a (*p* = 0.003), miR-155 (*p* = 0.004) and miR-181 (*p* = 0.023) in cancer patients with low VAT vs. controls and higher expression of these miRs in cancer patients with high VAT vs. controls (*p* = 0.011, *p* = 0.024, *p* = 0.012, respectively) **[(B)**, from left to right] Expression levels of miR-26a, miR-155 and miR-181 were higher in cancer patients with low SAT vs. controls (*p* = 0.004, *p* = 0.006 and *p* = 0.016, respectively) and were also significantly higher in cancer patients with high SAT vs. controls (*p* = 0.010, *p* = 0.019 and *p* = 0.018, respectively). **[(C)**, from left to right] Considering TAT, we observed that miR-26a, miR-155 and miR-181 were higher in cancer patients with low TAT vs. controls (*p* = 0.008, *p* = 0.001 and *p* = 0.022, respectively) and that miR-26a and miR-181a were higher in cancer patients with high TAT vs. controls (*p* = 0.004 and *p* = 0.012, respectively). ^*^ *p* < 0.05, ^**^ *p* ≤ 0.01, ^***^ *p* ≤ 0.001. Abbreviations: VAT, visceral adipose tissue; SAT, subcutaneous adipose tissue; TAT, total adipose tissue.

According to adiposity level (low vs. high median values of VAT, SAT, and TAT, resulting as follows: 130.70, 167.57 and 322.56 cm^2^), we observed for some miRNAs significant differences between cancer patients and controls (Figure 5). No differences were found between cancer patients with low vs. high adiposity (Figure 5).

**FIGURE 6:**
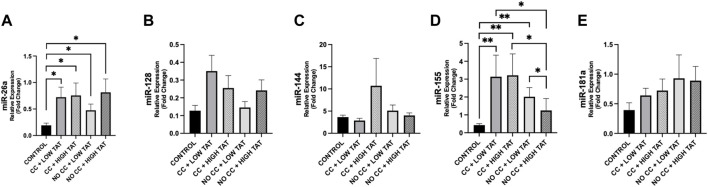
Differences in circulating miRNAs according to TAT in cachectic and non-cachectic cancer patients. We analyzed the circulating miRNAs according to the presence/absence of cancer cachexia and low/high levels of TAT (based on median value of 322.56 cm^2^) in cancer patients vs. controls. Groups are the following: cancer patients with cachexia and low TAT (n = 12); cancer patients with cachexia and high TAT (n = 6); cancer patients without cachexia and low TAT (n = 7); cancer patients without cachexia and high TAT (n = 12); controls (n = 14). **(A)** MiR-26a showed different modulation in cancer groups vs. controls (*p* < 0.05). **(D)** MiR-155 showed different levels between cancer groups and controls (*p* < 0.05) and we observed a significant downregulation of this miRNAs between cancer patients with cachexia and high TAT vs. cancer patients without cachexia and high TAT (*p* = 0.031) and higher levels in cancer patients without cachexia and low TAT compared to those with high TAT (*p* = 0.036). **(B,C,E)** No significance was observed in other miRNAs. ^*^ *p* < 0.05, ^**^ *p* ≤ 0.01, ^***^ *p* ≤ 0.001. Abbreviations: TAT, total adipose tissue; CC, cancer cachexia; NO CC, no cancer cachexia.

Analyzing the differences between cachexia/no-cachexia groups with low or high TAT, we observed that miR-155 was higher in cachectic patients with low TAT compared to those without cachexia and high TAT (*p* = 0.036) (Figure 6). Finally, miR-155 was higher in cachectic patients, regardless of low or high TAT, when compared to controls (Figure 6). Moreover, miR-155 expression was increased in cancer patients without cachexia with low TAT compared to those non-cachectic with high TAT (Figure 6). These differences were not present for the other miRNAs (Figure 6).

## 4 Discussion

Our study confirms the presence of clinical and nutritional changes in patients with gastrointestinal cancer undergoing surgery for cancer resection. In fact, although the main nutritional (anthropometric) parameter routinely used in clinic - BMI - was not different between cancer patients and controls, the presence of cachexia was highly prevalent (49%) in cancer patients. This is of particular interest taking into account that the patients of the present cohort were at their first cancer diagnosis, eligible to surgery and did not yet receive anticancer treatments. Our results indicate that patients satisfying the Fearon’s criteria for cachexia (Fearon et al., 2011) showed reduced SAT, suggesting the presence of adipose tissue metabolic derangements since the early phase of cancer disease. Interestingly, the phenomenon of adipose atrophy occurring in cancer was described even preceding the clinical diagnosis of pancreatic cancer (Babic et al., 2023). In another study, during the early phase of cancer disease, we described several histological alterations in SAT, including decreased adipocyte size, increased fibrosis and increased inflammatory infiltration in cachectic patients (Molfino et al., 2022b). Moreover, several evidence support that an increased lipolysis and browning of WAT are crucial in promoting cancer cachexia (Baracos et al., 2018; Hu et al., 2023; Geppert and Rohm, 2024).

Regarding the complex pathophysiology of adipose tissue changes and atrophy in cancer, the implication of circulating miRNAs has been recently supported by different studies (Di et al., 2021; Li et al., 2022; Hu et al., 2023). In our analyses, we found higher circulating levels of miR-26a, miR-128, miR-155 and miR-181a in cancer group compared to controls, and when observing the modulation of these miRNAs according to BMI classes, we found in patients with BMI < 25 kg/m^2^ that miR-26a was overexpressed compared to those defined as overweight or obese based on BMI ≥ 25 kg/m^2^, as well as to controls. No statistical difference was found for miR-128.

In line with our results, a recent study investigating circulating and visceral adipose miRNAs showed a downregulation of miR-26a in both plasma and VAT in patients with obesity compared with patients with normal weight, suggesting a potential role of this miRNA in regulating adiposity (Kim et al., 2020). However, in another study miR-128 overexpression was shown to play a regulatory role in adipose tissue metabolism, in particular by controlling adipogenesis and lipolysis (Chen et al., 2018). However, conclusive data on these miRNAs are still lacking in the literature.

In addition, we observed significant differences of miR-26 and miR-181a according to the presence of cachexia compared to controls, although the difference was also significant between non-cachectic and control groups. For this reason, at this time it is not possible to indicate a clear implication of these miRNAs in the pathophysiology of cancer cachexia. However, miR-155 showing a similar behavior of miR-26a and miR-181a tended to be modulated in cachectic when compared to non-cachectic patients, although this result should be verified in larger sample.

MiR-155 appears of particular interest in adipose tissue wasting based on the data showing a potential role in promoting derangements of adipose tissue metabolism, by the suppression of adipogenesis and promotion of browning process in gastrointestinal and breast cancers (Liu et al., 2022; Sun et al., 2023). Also, Yehia et al., showed upregulation of miR-155 in cachectic patients with lung and pancreatic cancer (Yehia et al., 2021).

Body composition analysis, specifically addressing the level of adiposity in our cancer group, revealed a significant reduction in terms of SAT between cachectic and non-cachectic cancer patients. The absence of difference in VAT is likely due to the early stage of cancer disease. Our observation appears interesting considering that other authors found that SAT is implicated in wasting condition during the early phase of catabolic status. In fact, Sah et al. observed in patients with pancreatic cancer a loss of SAT that started 18 months before the cancer diagnosis, anticipating visceral adipose tissue and skeletal muscle atrophy (Sah et al., 2019). Atrophy of SAT was at least in part associated with an increased expression of UCP1 in SAT representing a potential biomarker of early adipose tissue metabolic derangements (Sah et al., 2019), but no information on miRNA profiling in this setting was available.

In our study, when assessing the differences between cachectic patients with low or high TAT, we found that only miR-155 was higher in those with low TAT compared to patients without cachexia and high adiposity. This may suggest an implication of miR-155 in patients with high catabolic status.

Our study has limitations, including the type of gastrointestinal cancer patients, mainly represented by colorectal cancer. Cancer group was older than controls and this difference in age may affect adipose tissue distribution. The inclusion of other gastrointestinal tract cancer, in particular stomach and pancreas, may reveal additional miRNAs modulation that may occur in cancer and in cachexia. The level of adiposity was not assessed in control group and this information may be clinically relevant when comparing adipose tissue changes and its distribution and miRNAs profile in non-catabolic/non cancer conditions. However, CT scan for body composition analysis was not possible for ethical reasons in our controls. We selected specific miRNAs for adipose tissue metabolism, but Next-generation Sequencing (NGS) panel may allow for the identification of a larger number of miRNAs potentially involved in changes in adipose tissue. However, our methodological approach based on analyzing specific miRNAs known to be implicated in biological processes was previously implemented (Lin et al., 2022; Molfino et al., 2023b). The analysis in the subgroups (e.g., cachectic with low or high adiposity) was conducted in small number of participants and need further verification.

## 5 Conclusion

In the present study, we observed modulations in specific miRNAs involved in adipose tissue metabolism and cachexia in plasma of cancer patients, according to the presence of cachexia and low adiposity. Among others, miR-155 was shown to be modulated according to the presence of both cachexia and low adiposity evaluated by CT scan. To unveil the involvement of miRNAs in the pathophysiology of cachexia may allow for a prompt and early diagnosis of nutritional and metabolic alterations in cancer providing the rationale for innovative treatments.

## Data Availability

The raw data supporting the conclusions of this article will be made available by the authors, without undue reservation.
